# Potato (*Solanum tuberosum* L.) Plant Shoot and Root Changes under Abiotic Stresses—Yield Response

**DOI:** 10.3390/plants11243568

**Published:** 2022-12-17

**Authors:** Dominika Boguszewska-Mańkowska, Krystyna Zarzyńska, Beata Wasilewska-Nascimento

**Affiliations:** Plant Breeding and Acclimatization Institute—National Research Institute, Jadwisin, Szaniawskiego 15, 05-140 Serock, Poland

**Keywords:** *Solanum tuberosum*, drought and heat, root, shoot, yield

## Abstract

During the growing season, potato plants are often exposed to soil drought, frequently accompanied by heat stress, which results in crop losses. In our experiment, the impact of these stresses, both separately and simultaneously, on the above-ground, on the root, and on the tuber mass was assessed. Four potato cultivars were tested. In vitro plants were planted in plastic tubes. Four treatments were used: control–optimal irrigation and temperature (22/18 °C), drought stress, high temperature stress (38/25 °C), and drought and high temperature stresses combined. The stresses were applied for two weeks during the tuberization phase. Both stresses caused changes in plant morphology. Drought stress had a greater impact on these changes than high temperatures. The biggest changes, however, took place when both stresses were applied simultaneously. Under all stresses, a decrease in tuber yield was found. The largest decrease was recorded in the case of applying both stresses simultaneously, while the smallest one was in the case of high temperature stress in relation to a control condition. Among the morphological parameters studied, the mass of the root system and its share in the entire biomass of the plant had the greatest impact on the decrease in yield. This mainly concerned drought stress.

## 1. Introduction

Climate models predict that global warming will further escalate drought because of increasing evapotranspiration [1,2]. Land areas affected by extreme droughts are estimated to increase from 1% to 30% globally by 2100 [3]. There are likely to be large regional differences [4]. Simulation models (in the DSSAT package) showed a relatively uniform 1.6 °C increase in temperature in Ireland by 2075 [5,6]. Temperate regions are projected to experience more varied weather patterns, with northern Europe receiving more rainfall in the winter and much less during the summer [7]. Such changes will affect agricultural production. Drought is projected to reduce potential potato yields worldwide by 18–32% in the years 2040–2069 [8].

Heat and drought are two different types of abiotic stresses that can occur simultaneously or separately in the field.

High temperatures affect potato production. The research results show that soil temperature over 18 °C tends to reduce tuber yield, especially when combined with high ambient air temperature. Heat stress is responsible for the imbalance in the source-sink relationship, delays in tuber initiation and bulking, malformation, and necrosis of tubers [9]. Heat stress is defined as an increase in temperature above a threshold level for a period of time sufficient to cause irreversible damage to plant growth and development [10]. According to Wahid et al. (2007), transitory or constantly high temperatures cause a number of morpho-anatomical, physiological, and biochemical changes in plants, which affect their growth and development and may lead to a drastic reduction in economic yield.

Drought limits the productivity of crop plants by affecting the photosynthetic processes at the canopy, leaf, or chloroplast level, either directly, or by feedback inhibition if transport of photosynthate to sink organs is limited [11]. The response pattern of plants to drought stress depends on the intensity, duration, and rate of progression of severe drought [12,13].

The breeding of heat- and drought-tolerant potato cultivars is one of the most feasible and practical approaches to cope with global warming. However, breeders are generally focused on the development of either heat- or drought-tolerant potato cultivars, rarely considering tolerance to both stresses. Previous studies [14,15,16,17] indicate that the tolerance mechanism for heat and drought is different in potatoes. According to Wahid [10] and Haverkort and Verhagen [7], the adverse effects of heat stress can be mitigated by developing crop plants with improved thermotolerance using various genetic approaches. For this reason, it is necessary to thoroughly understand the physiological responses of potato plants to high temperatures [9].

Most publications focus on heat and drought stress on the above-ground part of the plant, the changes caused by those stresses, and their influence on tuber yield [10,18,19].

There is less information on the impact of both stresses on the size of the root system and its architecture [20].

The aim of our work was to assess the response of selected potato cultivars to high temperature and drought stresses, simultaneously and separately, to study morphological changes that occur in the above-ground part of plants, their impact on the size of the root system, and to find correlations between plant parameters and tuber yield under these stress conditions. Our working hypothesis assumes that the size of the root system in variety resistance to abiotic stresses is as important as the above-ground part of plants or even more important than that.

## 2. Results

### 2.1. Significance of Tested Parameters

The applied stresses resulted in significantly different values of all tested parameters (Table 1). Significant variation was observed between the cultivars in features such as: plant height, leaf dry mass, stem dry mass, and root dry mass, when all treatments were compared together. The significance of cultivar differentiation of leaf area and the share of roots in plant biomass has not been proven. The interaction of cultivars and treatments concerned the height of plants and dry mass of the stems. When comparing drought with the control condition or the simultaneous application of drought and heat with control conditions, cultivar significance was observed in the case of leaf dry mass, stem dry mass, and root dry mass. Additionally, cultivar significance of plant height was observed when comparing the control with high temperature conditions. The tuber yield depended significantly on the treatments used. No significance of cultivar diversity and interaction of cultivars with stress were found (Table 2).

### 2.2. Changes in the Morphological Plant Parameters

#### 2.2.1. Plant Height Depending on the Applied Stress and Cultivar Used

The applied stresses impacted the height of plants. On average for cultivars, plants growing under heat stress were significantly higher than under other treatments.

**Figure 1:**
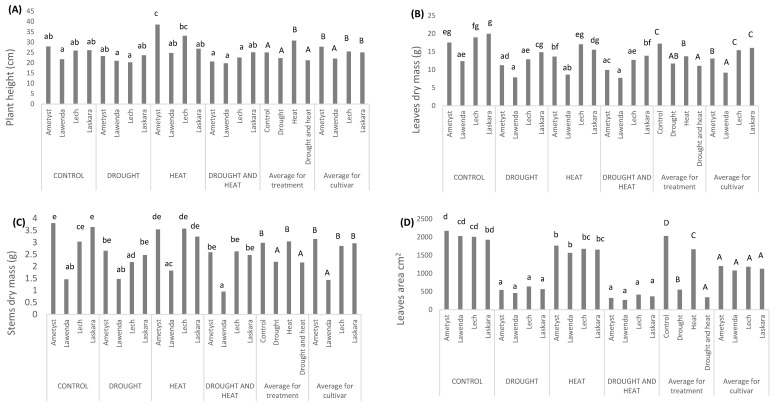

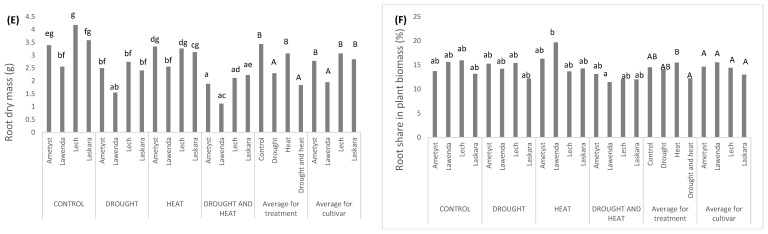
Average value of tested morphological parameters (**A**) plant height; (**B**) Leaves dry mass; (**C**) Stems dry mass; (**D**) Leaves area; (**E**) Root dry mas; (**F**) Root share in plant biomass; per plant of selected potato cultivars under drought, heat and both stresses applied simultaneously. a, b, c, A, B, C-values in the same column followed by the same letter do not significantly differ (*p* < 0.05), as per Tuckey’s test.

The observed average increase in plant height under high temperature stress was 23.1% compared to the control, and the decrease under drought stress and both stresses applied simultaneously was 11.1% and 15.2%, respectively. Among the different cultivars, a significantly lower plant height was observed in Lawenda (Table 1, Figure 1A).

#### 2.2.2. Leaf Dry Mass Depending on the Applied Stress and Cultivar Used

The applied stresses also significantly influenced the leaf dry mass (Table 2). On average, for cultivars, the highest dry mass of leaves was observed in the control treatment. The lowest leaf mass was noted in the treatment where both stresses were applied simultaneously. The decrease in dry mass of leaves compared to the control conditions was 31.9% under drought stress, 20.3% under high temperature stress, and 35.7% under both stresses simultaneously. The largest mass of leaves was noted for the Laskara and Lech cultivars, while the smallest mass was observed for Lawenda (Table 1, Figure 1B).

#### 2.2.3. Stem Dry Mass Depending on the Applied Stress and Cultivar Used

The applied abiotic stresses had a significant impact on the dry mass of the stems. The highest stem mass was recorded for the control and high temperature treatments. Dry mass of the stem was lower by as much as 26.5% and 27.5% compared to the control in the case of drought and after applying both stresses simultaneously. The Lawenda cultivar had the lowest dry mass of stems (Table 1, Figure 1C).

#### 2.2.4. Leaf Area Depending on the Applied Stress and Cultivar Used

The applied stresses compromised the leaf area of plants the most. When comparing the leaf area, significant differences between the treatments were noted. The control plants had the largest area. This decreased by 72.9% and 83.1%, respectively, in plants subjected to drought stress and both stresses combined. The leaf area of plants exposed to high temperature decreased by 18.1% compared to the control. The tested cultivars did not differ significantly in leaf area (Table 1, Figure 1D).

#### 2.2.5. Root Dry Mass Depending on the Applied Stress and Cultivar Used

A significant decrease in the dry mass of the root system in relation to the control treatment occurred in the case of drought stress and both stresses applied simultaneously. The decrease was 32.9% and 46.4%, respectively.

Under high temperature stress, the mass of the root system was not insignificantly lower (compared to control) (Figure 1E). High temperature stress caused a root mass decrease of 10.5%. There was a significant difference in the size of the root system between the Lawenda cultivar and others. This cultivar showed the lowest dry mass of roots (Figure 1E).

#### 2.2.6. Root Share in Plant Biomass Depending on the Applied Stress and Cultivar Used

The applied stresses significantly influenced the share of roots in the entire biomass of the plant. A significantly lower share of roots in total biomass was found after applying both stresses simultaneously. The decrease in this case was 15.7%. In the heat treatment, the share of roots in total biomass was the highest and exceeded the control by 6.7%. In the control, the share of roots was higher than in the drought treatment, but the differences were not significant (Figure 1F). No cultivar differences have been proven for this feature.

### 2.3. The Impact of Applied Stresses on the Tuber Yield

The stress conditions significantly impacted the tuber yield (Table 2). On average, for cultivars in the control treatment, the tuber yield was 1440 g. Under all stresses, there was a decrease in yield, but it varied depending on the stress applied. The lowest decrease in yield in relation to the control occurred after applying high temperature stress (14.9%), a bigger decrease was observed under drought stress (23.2%), while the highest decrease occurred when both stresses were applied simultaneously (29.2%). There were no significant cultivar differences.

### 2.4. The Relationships between Selected Morphological Indicators of Plants and Yield Decrease under Stresses

Based on the data presented, correlation coefficients between morphological indicators and yield decrease under stress (when compared to controlled conditions) were calculated for drought and heat stresses separately and for both of these conditions simultaneously.

In drought stress, a significant relationship formed between the root dry mass, the root share in biomass, and the plant height. In the case of root mass, there were inverse correlations, i.e., the smaller the mass of the roots and the smaller their share in the plant biomass, the higher the decrease in yield. In the case of plant height there was a direct relationship. No significant relationships were found for the mass of leaves, mass of stems, and plant leaf area (Table 3).

Similar relationships were noted in the treatment with high temperature stress, but in no case was the relationship statistically significant (Table 4).

The highest correlations were obtained in the treatment where the two stresses were applied simultaneously. Significant correlations were observed between the root mass, the root share in plant biomass, and the plant height. In the case of the mass and share of roots in biomass there was an inverse correlation, whereas the plant height formed a positive correlation, i.e., the higher the plants, the higher the decrease in yield. However, the relationship between plant height and yield decline was weaker than between the root system size and yield decline (Table 5).

## 3. Material and Methods

### 3.1. Plant Material

The study was carried out at the Plant Breeding and Acclimatization Institute-National Research Institute in Poland. Four ware potato cultivars were tested: Ametyst, Laskara, Lech (mid early), and Lawenda (early). Average yield according to the Polish Cultivar Register [21] is 64.4 t/ha, 55.2 t/ha, 44.3 t/ha, and 49.7 t/ha, respectively. All cultivars originated from Polish potato breeding stations.

### 3.2. Tube Experiment

In vitro plants were planted in plastic tubes with a diameter of 12 cm and a length of 50 cm. Tubes were filled with soil composed of light loamy sand brought from the field where potatoes are grown and mixed with sand in a ratio of 1:1. During the growing season, plants were fertilized with Yara Mila Viking NPK 14-14-21 with a dose of N = 2.1 g, *p* = 1.0 g, K = 2.4 g, and Mg = 0.4 g per plant.

Plants were grown in controlled conditions in vegetation chambers (22 °C/18 °C—14 h day/10 h night). To maintain optimal water condition, that is 70% of field water capacity, plants were watered daily by a drip irrigation system. The water field capacity was measured with a soil moisture tester (10HS ECH20 METER Group, Inc., Pullman, WA, USA). The vegetation chamber was equipped with six Hortilux Schreder Lamps with Philips light bulbs of 1600 W each. Air humidity was in the range 65–70%.

During the flowering period (60–65 BBCH), the following treatments were used for two weeks:

- control-optimal irrigation (70% of field water capacity) and optimal temperature (day/night temperature 22/18 °C);
- drought stress-remained without irrigation (40% of field water capacity) and optimal temperature (day/night 22/18 °C);
- high temperature stress-optimal irrigation (70% of field water capacity) and maintenance of elevated temperature (day/night temperature 38/25 °C);
- combined drought-high temperature stress-remained without irrigation (40% of field water capacity) and maintenance of elevated temperature (day/night temperature 38 °C/25 °C).

The experiment was carried out two times. Each time, six plants were tested for each treatment. After the application of stresses, the following measurements of potato plants were taken: height (cm), dry mass of stems (g), dry mass of leaves (g), leaf area (cm2), dry mass of roots (g), and share of roots in total biomass. In order to determine the dry mass, individual plant organs were dried in two stages: 24 h drying at 75 °C and then at 105 °C until the weight stabilized. Leaf area was measured with an LI-3100A (LI-COR, Pullman, WA, USA) instrument [22].

### 3.3. Pot Experiment concerning the Yield

The crop yielding was also assessed in a control conditions-vegetation chamber (22 °C/18 °C)-14 h day/10 h night). Plants of four of the same cultivars: Ametyst, Laskara, Lech, and Lawenda were grown in 12 l pots filled with a thin layer of gravel at the bottom and universal vegetable soil substrate ‘Hollas’, which is produced from peat with the addition of chalk at a pH range of 5.5–6.5. For improved soil aeration, a gum pipe was installed in each pot.

Tubers of 3–4.5 cm in diameter were selected for planting. Two weeks before planting, high-quality seed potatoes were subjected to pre-sprouting and then ploughed into the pot soil at a depth of 5–6 cm. Plants were watered daily with an optimal tap water supply that is 70% of field water capacity. The water field capacity was monitored using a soil moisture tester 10HS ECH20 METER Group, Inc. (Pullman, WA, USA).

During the flowering period (60–65 BBCH), the same four treatments as in the tube experiment were used for two weeks: control, drought stress, high temperature stress, and combined drought-high temperature stress. After the end of the growing season, tuber yield (g) was measured from each treatment.

Statistical analyses of the results were performed with an analysis of variance and regression using Statistica 13.3 program (StatSoft, Poland). The significance of the sources of variation was tested with a Fisher-Snedecor test, and the significance of differences was assessed using Tukey’s test.

## 4. Discussion

The potato is particularly susceptible to both drought [23] and heat [9] stresses. Drought susceptibility of the potato has been mainly attributed to an inability to capture water, given its shallow root system and an inability of the photosynthetic machinery to recover following both water and heat stresses [24]. In our research, the impact of both stresses separately and simultaneously was assessed. The experiment was focused mainly on changes concerning the above-ground part of the plant as well as the root system under the influence of selected abiotic stresses.

According to many authors, the reaction of potato plants to both stresses is different [6,15,16,17,25]. Our research confirmed the varied reactions of potato plants to both stresses. The drought stress caused much larger changes in the size of both the shoot and the root, compared with high temperature stress. Water stress studies on potatoes have shown a reduction in expansion of stems and leaves, leading to reduced foliage, reduced canopy, reduced leaf area index, decreased shoot biomass, and finally reduction in dry matter content [15,26,27,28,29]. In our research, drought stress inhibited plant growth, as was evidenced by the leaf dry mass, stem dry mass, leaf assimilation area, and root dry mass. High temperature stress resulted in the lengthening of plants but decreased weight of leaves; plants were higher but thinner.

The sum of stresses caused the biggest changes, but the bigger impact of drought over heat stress was clearly visible in this study. Crop loss due to drought and heat stress was bigger than each of the single stresses separately [30]. The co-occurring drought and heat stress should be of serious concern since this phenomenon is getting worse, as climate change causes an increase in the average earth surface temperature and low precipitation in some areas [31,32]. However, information about the simultaneous impact of drought and heat stresses on potato plants is rare. Handayani and Watanabe [19] assessed two stresses separately and together and confirmed that the combined drought-heat stress had a greater effect on the tested traits.

The morphological plant responses depend on the timing of stress application (development stage), and also on whether the stress is short or long term. Stresses applied during the tuber initiation and tuberization stages not only restrain foliage and plant development, but also limit tuber mass and, therefore, reduce the harvest index (HI) and dry matter with effects on final yield [14,18]. In our research, yield decrease affected all tested cultivars. From the agricultural practice point of view, the reaction of individual cultivars to both stresses is important [13,17,33]. In our research, the Lawenda cultivar had the highest yield decrease under drought and combined stresses, as well as the highest levels of morphological changes. A smaller yield decrease under drought stress was noticed in both Lech and Ametyst cultivars. The cultivar that exhibited the lowest yield decrease under the influence of high temperature stress was the Lech cultivar.

Thus, the diversified varietal response in the development of the above-ground part of the plant was reflected in the yield decrease under the applied stresses. Even greater differences were noted in the case of root mass. As it is known, the root system plays a very important role in the resistance of plants to drought stress. Bigger root systems in potato genotypes, particularly those roots associated with stolons, lead to a better ability to cope with drought and maintain yield under drought conditions [34,35,36]. Interestingly, genotypes with bigger roots systems also achieved a quicker canopy closure, which extends the duration of maximal photosynthetic potential and more quickly reduces the amount of water lost from the soil by evaporation, rather than transpiration through the plant. It has been suggested that breeding for the number of seminal roots, root hair length, and increased root hydraulic resistance is possible [37]. In our research, the greatest mass of roots was found in the Lech cultivar, which was also characterized by a high shoot mass.

Root system studies are very difficult and time-consuming to perform, especially with the use of abiotic stresses. The results from model tests cannot always be directly transferred to field conditions, but they constitute valuable information for the breeding process. The stress of drought reduces the mass of the root system, which has been confirmed by Boguszewska-Mańkowska et al. [20], Lahlou and Ledent [38], and Zarzyńska et al. [39]. Additionally, extreme heat leads to root growth inhibitions [6]. Our research also showed a reduction in the root mass under high temperature stress, but there were significantly smaller differences than in the case of drought stress.

The genetic diversity of cultivars, regarding their tolerance to abiotic stresses, is well known. They also concern the size of the root system. Such information is confirmed by the works of Boguszewska-Mańkowska et al. [20], Iwama [24], and Zarzyńska et al. [39].

In our experiment, the size of the root system had the biggest role in the decrease of tuber yield, mainly under drought stress. In drought conditions, despite the decrease in both the above-ground mass and the mass of the root system, the share of roots in the total biomass remained at a similar level. The tendency even for an increased root–shoot ratio under drought and for the prioritization of root growth over shoot growth [20,40] are both likely to contribute to drought tolerance. The positive correlation between root mass, shoot mass, and final tuber yield have led to the suggestion of using root mass in the lower layers as a selection criterion [24].

It is noteworthy to mention that the concept of root ideotype can only be exploited in practical field breeding with a thorough knowledge of the plant’s stress environment as well as the metabolic cost sustained by the plant to develop and maintain a more vigorous root system [25]. In addition to the well-established factors of root density and depth, the hydraulic characteristics of the plant, and its interaction with the soil environment is highly significant in drought adaptation [41].

## 5. Main Conclusions

1. Drought and high temperature, both separately and together, caused changes in plant morphology. Drought stress had a greater impact than high temperature stress. The biggest changes, however, took place when both stresses were applied simultaneously.
2. Under all stresses, a decrease in tuber yield was found. The largest decrease was recorded in the case of both stresses applied simultaneously. The smallest decrease was visible in the case of high temperature stress.
3. The greatest impact on the decrease in yield under the applied stresses was due to the root system size and its share in the entire biomass of the plant. This impact was higher in the case of drought stress than in heat stress.
4. Our research confirms the importance of the root system size in the resistance of potato cultivars to abiotic stresses, and in particular to drought stress, and may be a valuable clue for potato growers.

## Figures and Tables

**Table 1:** Sources of variation, ANOVA results and levels of significance (*p*-values) for treatments affecting tested parameters.

Tested Stress	Sources of Variation	Plant Height (cm)		Leaves Dry Mass (g)		Stems Dry Mass (g)		Leaves Area (cm^2^)		Root Dry Mass (g)		Root Share in Plant Biomass (%)	
		*p*-Value	Significance	*p*-Value	Significance	*p*-Value	Significance	*p*-Value	Significance	*p*-Value	Significance	*p*-Value	Significance
Drought	Cultivar	0.4991	-	0.0000	***	0.0000	***	0.548	-	0.0091	*	0.3953	-
	Treatment	0.1292	-	0.0000	***	0.0000	***	0.0000	***	0.0000	***	0.2521	-
	Treatment × cultivar	0.5820	-	0.784	-	0.0981	-	0.3681	-	0.7152	-	0.9790	-
Heat	Cultivar	0.0026	*	0.000	***	0.000	***	0.694	-	0.0356	*	0.3791	-
	Treatment	0.0033	*	0.000	***	0.776	-	0.000	***	0.0491	*	0.6412	-
	Treatment × cultivar	0.3701	-	0.802	-	0.328	-	0.883	-	0.6344	-	0.4412	-
Drought & Heat	Cultivar	0.3231	-	0.0000	***	0.0000	***	0.6502	-	0.0085	*	0.5102	-
	Treatment	0.1428	-	0.0000	***	0.0003	**	0.0000	***	0.0000	***	0.0466	*
	Treatment × cultivar	0.5971	-	0.6491	-	0.2502	-	0.3751	-	0.8293	-	0.7702	-
Drought Heat, Drought & Heat	Cultivar	0.0000	***	0.0000	***	0.0000	***	0.148	-	0.0000	***	0.3471	-
	Treatment (Control, Drought, Heat, Drought & Heat)	0.0010	**	0.0000	***	0.0000	***	0.000	***	0.0000	***	0.0071	*
	Treatment × cultivar	0.0120	*	0.7721	-	0.0391	*	0.678	-	0.7271	-	0.1670	-

* significant at α = 0.05; ** significant at α = 0.01; *** significant at α = 0.001; - not significant.

**Table 2:** Tuber yield (g/plant) depending on the applied stress, potato cultivar and the yield decrease (%) in relation to the control.

Cultivar/Treatment	Control	Drought	Decrease (Relative to Control)	Heat	Decrease (Relative to Control)	Drought and Heat	Decrease (Relative to Control)
Ametyst	1467 ^c^	1184 ^a,b^	19.3	1263 ^b^	13.9	1120 ^b^	23.6
Laskara	1397 ^b,c^	1033 ^a,b^	26.0	1128 ^b^	19.2	955 ^a^	31.6
Lawenda	1433 ^c^	1027 ^a,b^	28.3	1184 ^b^	17.3	959 ^a^	33.1
Lech	1465 ^c^	1184 ^a,b^	19.2	1330 ^b,c^	9.2	1045 ^a,b^	28.6
Mean	1440 ^C^	1107 ^A,B^	23.2	1226 ^B^	14.9	1020 ^A^	29.2

^a^, ^b^, ^c^, ^A^, ^B^, ^C^-values in the same column followed by the same letter do not significantly differ (*p* < 0.05), as per Tuckey’s test.

**Table 3:** Correlation coefficients between the selected morphological parameters of potato plant and yield decrease in relation to the control under drought stress.

Tested Correlation	*p*	r	r^2^
Yield decrease-plant height	0.0393	0.0144	0.0002
Yield decrease-leaves dry mass	0.3190	0.2122	0.0450
Yield decrease-stems dry mass	0.7869	−0.00582	0.0034
Yield decrease-leaves area	0.2090	0.2656	0.0430
Yield decrease-root dry mass	0.0006	−0.6478	0.4190
Yield decrease-root share in plant biomass	0.0012	−0.620	0.3850

**Table 4:** Correlation coefficients between the selected morphological parameters of potato plant and yield decrease in relation to the control under heat stress.

Tested Correlation	*p*	r	r^2^
Yield decrease-plant height	0.9466	−0.0656	0.0043
Yield decrease-leaves dry mass	0.9750	−0.0068	0.0000
Yield decrease-stems mass	0.4882	0.1487	0.0221
Yield decrease-leaves area	0.4450	0.1636	0.0268
Yield decrease-root dry mass	0.0734	−0.0732	0.00054
Yield decrease-root share in plant biomass	0.6316	−0.1031	0.0136

**Table 5:** Correlation coefficients between the selected morphological parameters of potato plant and yield decrease in relation to the control under drought and heat stresses applied simultaneously.

Tested Correlation	*p*	r	r^2^
Yield decrease-plant height	0.0266	0.4521	0.2044
Yield decrease-leaves dry mass	0.1409	0.3096	0.0959
Yield decrease-stem dry mass	0.1415	0.3092	0.0956
Yield decrease-leaves area	0.0926	0.3511	0.1232
Yield decrease-root dry mass	0.0009	−0.6350	0.4032
Yield decrease-root share in plant biomass	0.0001	−0.6989	0.4885

## Data Availability

The datasets generated during and/or analyzed during the current study are available from the corresponding author on reasonable request.
